# DNA Metabarcoding Reveals the Diet and Conservation Needs of the Chinese Crested Tern and Greater Crested Terns in Zhejiang Breeding Islands

**DOI:** 10.1002/ece3.73492

**Published:** 2026-04-16

**Authors:** Peng Ding, Xilai Zhou, Siyu Wang, Yiwei Lu, Zhiwen Yan, Cheng Qian, Keying Zhu, Xiaopin Ni, Erwei Wu, Zhongyong Fan, Ke He

**Affiliations:** ^1^ Xiangshan Bureau of Natural Resources and Planning Zhejiang China; ^2^ College of Animal Science and Technology, College of Veterinary Medicine Zhejiang Agriculture and Forestry University Hangzhou China; ^3^ Zhejiang Museum of Natural History Hangzhou China; ^4^ Zhejiang Biodiversity Institute Hangzhou China; ^5^ Bird Branch of Zhejiang Wildlife Conservation Association Hangzhou China; ^6^ Administration of Nanji Islands National Marine Nature Reserve Wenzhou China; ^7^ Hangzhou Birds and Ecology Research Society Hangzhou China

**Keywords:** conservation, diet, DNA metabarcoding, fish, seabird, *Thalasseus bernsteini*

## Abstract

As top predators and environmental sentinels in marine ecosystems, seabirds face significant pressure from global fisheries in competing for fish resources. For the critically endangered Chinese crested tern ( *Thalasseus bernsteini* ), characterizing its dietary requirements is a fundamental step in assessing how food availability and potential fishery‐induced resource shifts may impact its recovery. This study, conducted on breeding islands (Xiangshan island and Nanji Island) in Zhejiang, analyzed *12S* and *COI* regions from fecal samples (*n* = 50) using DNA metabarcoding technology to systematically analyze the diets of mixed‐colony terns (Chinese crested terns and greater crested terns [ *T. bergii* ]) across the breeding season (breeding stage and nestling stage). The results for both species combined revealed a highly specialized diet, primarily focused on epipelagic schooling fish, with Engraulidae (occurrence frequency, FO = 72.73%) and Scombridae (FO = 27.27%) being the dominant taxa (combined relative read abundance, RRA > 54%). The key species were *Engraulis japonicus* , *Setipinna tenuifilis* , and *Thryssa kammalensis* . Our multi‐scale taxonomic analysis revealed that the dietary structure of these terns was conserved at higher taxonomic levels but highly dynamic at the species level. PCoA indicated no significant segregation between breeding stages or islands at the family level, whereas distinct, significant differences emerged between breeding stages at the species level, with nestling‐stage diets exhibiting greater diversity (significant higher Chao index), reflecting increased flexibility in resource utilization during the provisioning period. By integrating our findings with data from Australia and South Africa, we identify a universal foraging characteristic of *T. bergii* and *T. bernsteini* as a specialist predator primarily dependent on epipelagic schooling fish across its global range. Although the waters surrounding the study islands serve as an important spawning ground for various commercial fish species, the diet of these terns contains economically valuable fish in very low proportions (relative sequence abundance < 5%), indicating niche separation from direct fishery competition. Additionally, the first detection of freshwater fish in 6.82% of samples (3 out of 44) in the tern diet within this study area highlights the behavioral plasticity of terns in human‐modified environments and the potential for novel human‐bird conflicts at these sites. Our study emphasizes that securing these shared prey resources, particularly Engraulidae, is essential for the breeding success of these mixed colonies. Conservation strategies must extend beyond traditional habitat protection to incorporate coastal human activities into risk management frameworks, thereby enhancing the recovery of the critically endangered Chinese crested tern by protecting its primary food base.

## Graphical abstract

This study employs DNA metabarcoding to analyze the diet composition of the critically endangered Chinese crested tern and the greater crested tern during their breeding season in Zhejiang. The terns primarily consume epipelagic schooling fish like anchovies and mackerel, with minimal overlap with commercial fisheries (< 5% commercial fish). The detection of freshwater prey indicates behavioral adaptability to human‐altered coastal environments.

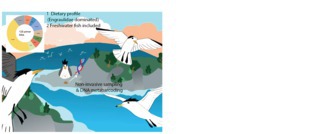

## 1 Introduction

As key predators in marine ecosystems, seabirds are estimated to consume 57–96 million tons of prey globally annually (Grémillet et al. 2018). Due to this high reliance on marine productivity, migratory seabirds, such as terns, can serve as sentinels of marine ecosystem changes, with their demographic traits, behavior, and physiological conditions reflecting shifts in marine environmental and ecological conditions (Hazen et al. 2019). For instance, temporal variations in the timing, abundance, and size of seabird prey can act as indicators of food availability and ecosystem structure (Parsons et al. 2008). As demonstrated by multi‐decadal time‐series data, these dietary shifts reflect cumulative environmental and anthropogenic pressures on marine food webs (Green et al. 2014), providing vital insights that are often missed by short‐term monitoring. Environmental variables influence local prey distribution and abundance. For example, sea surface temperature anomalies, such as marine heatwaves, can disrupt forage fish communities and force seabirds to extend their foraging ranges or switch prey, ultimately modulating their breeding success (Jones et al. 2018; Osborne et al. 2020; Watanuki et al. 2022). Therefore, monitoring seabird diets serves as a vital tool in conservation biology and ecosystem‐based fishery management by providing high‐frequency feedback on fish stock health, which allows managers to adjust fishing pressure in response to both natural environmental shifts and human activities (Parsons et al. 2008; Hazen et al. 2019; Velarde et al. 2019).

Seabirds worldwide exhibit altered behavior, energy metabolism, demographic traits, and population dynamics in response to various fisheries activities (Sherley et al. 2018; Votier et al. 2023). Seabird diets are highly diverse, characterized by a wide array of foraging guilds and prey types, including mollusks, crustaceans, and fish (Evans et al. 2025; Hernández and Arroyo 2025). While many piscivorous species primarily target small schooling fish such as sandeels, herring, and anchovies, this dietary composition varies significantly across species and foraging strategies (Rountos et al. 2015). For seabird species with a high dependency on specific forage fish, harvesting these populations may reduce prey availability below thresholds critical for their reproduction and survival (William et al. 2017). Reduced prey availability is recognized as a key factor in the decline of some seabird species at both local and global population scales, such as the Atlantic puffin (Fayet et al. 2021) and black‐browed albatross ( *Thalassarche melanophris* ) (McInnes et al. 2017). Fishing for low‐trophic‐level species can also negatively affect seabirds by triggering a “junk food” effect, where high‐energy prey is replaced by less nutritious alternatives (Whitfield 2008). A meta‐analysis of global seabird responses suggests that to maintain long‐term breeding success, approximately one‐third of the maximum prey biomass must be reserved to meet the energy demands of seabird populations (Cury et al. 2011). On a regional scale, this competitive relationship manifests as a significant spatial overlap between fishery activities and the distribution of specific seabird species, such as the yellow‐eyed penguin ( *Megadyptes antipodes* ); such overlap exerts observable direct impacts on their populations (Hickcox et al. 2023). For instance, the total prey biomass consumed by seabirds in Ireland is estimated to be on a scale equivalent to commercial fishery landings, with a high spatial overlap between seabird foraging activity and commercial fishing effort in coastal zones (Jessopp et al. 2025). Beyond resource competition, specific fishing methods like trawl fisheries pose direct risks through incidental bycatch, such as net entanglement and collisions with trawl cables (warps) (Phillips et al. 2024).

Fisheries‐seabird interactions are complex, characterized by both competitive and auxiliary roles. While direct competition for forage fish can drive breeding failure in specialized species (Cook et al. 2014; Searle et al. 2023), many seabirds exhibit behavioral plasticity by exploiting anthropogenic food subsidies (Vilaplana et al. 2024). For instance, species ranging from generalist scavengers to opportunistic foragers have adapted by synchronizing activities with fishing vessels or utilizing fishery discards as a dietary buffer against natural prey fluctuations (Votier et al. 2023; Vilaplana et al. 2024). Consequently, as global marine indicators, seabird populations face a multitude of global pressures, including climate change, invasive species at breeding colonies, and complex interactions with commercial fisheries (Dias et al. 2019; Votier et al. 2023).

Therefore, in‐depth dietary research into how these species utilize resources is essential for quantifying their overlap with commercial fisheries, thereby mitigating competition and informing ecosystem‐based management. Identifying specific prey allows conservationists to better evaluate conflicts with human activities, providing the critical evidence needed for targeted management and the protection of endangered seabird populations. Investigating dietary habits requires reliable research methods. Traditional approaches, such as stomach content analysis, are often invasive and can cause stress or harm to study subjects. Alternative non‐invasive methods, like identifying regurgitated pellets, avoid these welfare issues but frequently suffer from low taxonomic resolution and significant sampling bias, particularly due to the rapid digestion of soft‐bodied prey that leaves only hard structures (otoliths or beaks) detectable (McInnes et al. 2017; O'Hanlon et al. 2025). Direct observation is feasible for beak‐carrying species, such as terns, but may not cover the entire breeding cycle and can exclude small or easily digestible prey (Gaglio et al. 2018). In recent years, DNA metabarcoding technology, which analyses prey DNA in diverse biological samples (e.g., feces, pellets, and buccal swabs), has emerged as a powerful tool for studying seabird diets, offering high taxonomic precision in identifying a wide range of prey, including soft‐bodied organisms. This technique has been successfully applied to various seabirds (Pearson 2024; Siddiqi‐Davies et al. 2025), including terns (Cheng et al. 2025), enabling non‐invasive and detailed dietary profiling at the population level. Although this method is also primarily restricted to the breeding season due to the challenges of obtaining samples away from the colony, it allows for collection throughout the entire breeding period, offering a broader temporal window than visual observations, which are largely limited to the chick‐provisioning stage.

This study focused on the critically endangered Chinese crested tern ( *Thalasseus bernsteini* ) and the greater crested tern ( *T. bergii* ), which are typical sympatric, mixed‐colony breeding seabirds with strong morphological, behavioral, and ecological similarities (Chen et al. 2011). Among the six global breeding islands of the Chinese crested tern, most host mixed colonies with the greater crested tern, and 80% of the population breeds on the Zhejiang Islands, China (Hung et al. 2019; Lu et al. 2020). Beyond historical threats such as illegal egg collection and the synergistic effects of climate change (Chen et al. 2015), food availability is a key factor determining the population size of the Chinese crested tern. Early observations indicated that it primarily relied on fish, such as anchovies (Family Engraulidae, primarily *Engraulis japonicus* ), near breeding sites (Chen et al. 2011). As a key forage fish and mid‐trophic functional group, anchovies are essential for energy flow in the East China Sea, although the regional ecosystem faces significant pressure from overfishing (Cheng et al. 2009). Consequently, the colony size of terns is significantly correlated with local forage fish biomass (Roux 2006). Delays in breeding timing often subject parental birds to the dual pressures of adverse weather and food shortages, severely reducing reproductive success (Hung et al. 2019). Therefore, identifying the specific dietary composition of these birds is a prerequisite for protecting the productivity of key foraging grounds and fish resources, which is vital for the survival of this critically endangered population.

Therefore, this study focused on sympatric and mixed‐colony breeding populations of Chinese crested terns and greater crested terns on breeding islands (Xiangshan and Nanji) in Zhejiang Province. The Chinese crested tern was first recorded in Xiangshan in 2004. An artificial attraction and restoration program was initiated in 2013 and has since established stable breeding colonies of both Chinese crested tern and greater crested tern. In 2013, the colony comprised over 3300 greater crested terns and 19 Chinese crested terns. In recent years, the number of breeding Chinese crested terns recorded in the Xiangshan Islands reached 41 individuals in 2025; notably, a peak of 109 individuals was observed soaring over the islands in 2024. The Nanji Islands initiated an artificial attraction project in 2023, which successfully drew over 5000 terns by 2025, including a maximum single‐day count of 19 Chinese crested terns among the larger colony of greater crested terns. By collecting fecal samples from different breeding stages and using DNA metabarcoding methodology, we aimed to investigate their dietary structure during the breeding period. Specifically, we examined: (1) the dietary composition and structure of breeding terns in Zhejiang, and whether adult self‐feeding diets differ significantly before versus after breeding; and (2) whether the diet of breeding terns includes commercial fish species, potentially indicating conflicts between fisheries and tern conservation?

## 2 Material and Methods

### 2.1 Study Area and Sample

This study was conducted within the Xiangshan Islands National Nature Reserve (122°09′18″ E–122°15′24″ E, 29°22′30″ N–29°28′36″ N) and Nanji Islands National Nature Reserve (27°27′ N, 121°25′ E), both located in Zhejiang Province, China, and are separated by a distance of approximately 245 km.

**FIGURE 1:**
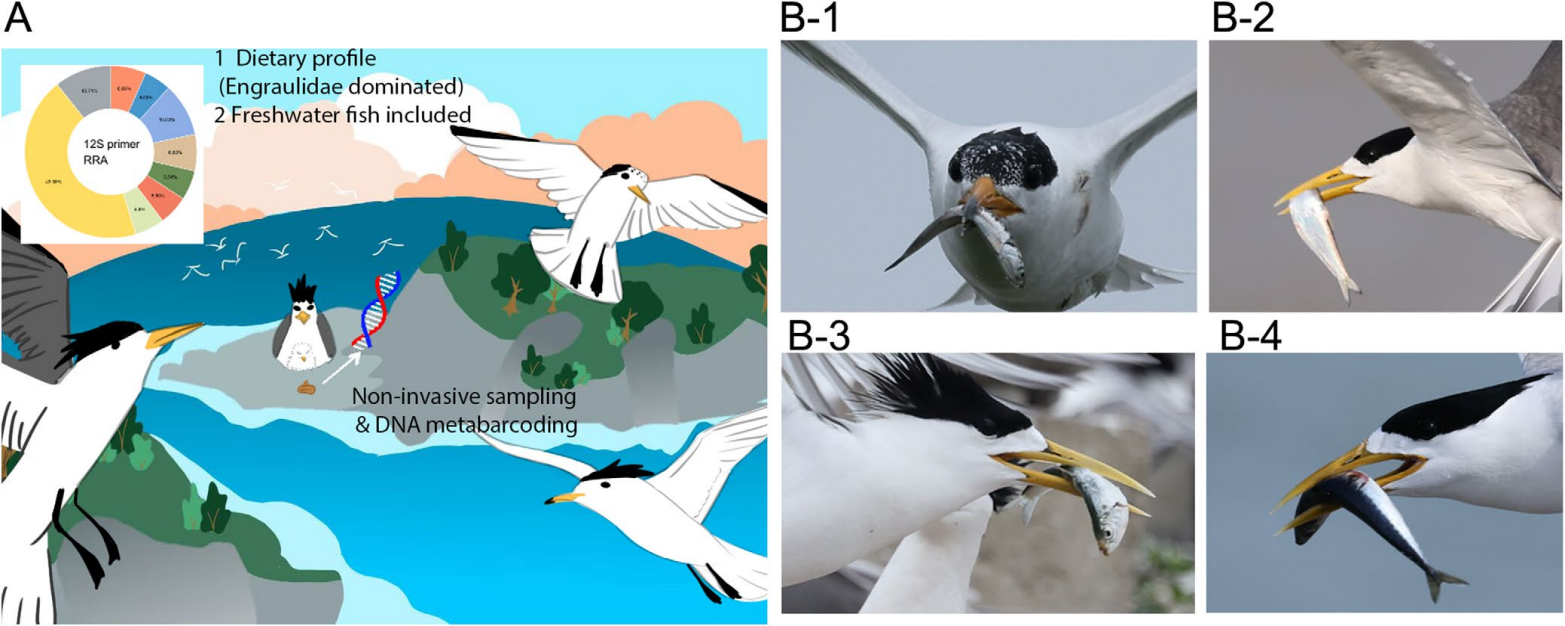
(A) The graphic abstract of this study, (B) Common fish prey recorded in the field monitoring, (B1) *Engraulis japonicus* , (B2) *Setipinna tenuifilis* , (B3) *Sardinella zunasi* , (B4) *Scomber japonicus* . Photo credit: Siyu Wang.

Field observations and fecal sample collection were conducted over seven sessions between May 25 and July 2, 2025 (Figure 1). This period coincided with the annual fishing moratorium (May 1 to September 16), ensuring that dietary data reflected natural foraging without influence from fishery discards. Based on real‐time monitoring of hatching individuals (June 10–15), we categorized the sampling into two stages: breeding (early May to June 8, covering courtship and egg production) and nestling (June 25 to July 2, focusing on chick‐rearing).

Fecal samples were obtained via two methods. For routine collection, cardboard sheets were placed at common perching sites. To ensure sample independence and prevent cross‐contamination, only single, distinct fecal droppings spaced at least 20 cm apart were collected. The time between excretion and collection did not exceed 12 h, for the breeding site was equipped with real‐time monitoring cameras. For the banding project, sterile cotton swabs were used to collect fecal material directly from the birds' cloaca under institutional ethical approval [Permit from Zhejiang Forestry Bureau 2025‐4 and 91]. All samples were immediately preserved in fecal DNA preservative (Simgen) in the field, stored at −20°C on island, transported to the laboratory at 4°C, and stored at −80°C until DNA extraction.

Fifty samples were collected: 34 from routine collections and 16 during banding (Table 1). For banded individuals, species identity was confirmed “in the hand” by experienced researchers using diagnostic traits (e.g., bill coloration). It is acknowledge that routine ground samples likely originated from greater crested terns due to their higher population density. Hybridization occurs at an extremely low frequency in these colonies, and no individuals with intermediate morphological characters were observed during our study. In summarize, five samples originated from Chinese crested terns, while the remainder (*n* = 45) were from greater crested terns. Notably, only three samples were collected from the Nanji Islands during the nestling stage.

**TABLE 1:** Sample information of this study.

Sampling site	Date	Stage	Method	Samples number	Effective samples	
					12S	COI
Xiangshan	25 May to 8 June	Breeding	Routine sampling	6	5	0
	31 May	Breeding	Banding collection	6	4	2
	25 June to 2 July	Nestling	Routine sampling	28	27	12
Nanji	24 May	Breeding	Banding collection	7	6	1
	2 July	Nestling	Banding collection	3	2	2

*Note:* Effective samples' refers to the number of fecal samples that remained for downstream analysis after quality filtering, denoising, and the removal of sequences with insufficient depth or non‐target prey taxa.

### 2.2 DNA Metabarcoding Sequencing and Analysis

#### 2.2.1 High‐Throughput Sequencing

During long‐term routine monitoring (daily 1‐h visual observations via binoculars, weather permitting), the terns' primary diet consisted of fish with a small proportion of marine arthropods (observed in our field survey). Therefore, we used two primer sets for amplification, a fish‐specific primer set and a metazoan primer set, to comprehensively cover the potential dietary spectrum of terns. For fish detection, the MiFish‐U primer pair was used, which specifically targets the 12S rRNA gene region and efficiently detects various fish components in the diet (Miya et al. 2015). In other marine organisms, the mlCOIintF/jgHCO2198R primer pair has been used to amplify specific segments of the cytochrome c oxidase subunit I (*COI*) gene (Geller et al. 2013; Leray et al. 2013). DNA metabarcoding sequencing was conducted by Majorbio Technology Co. Ltd. (Shanghai, China) using the Illumina Nextseq2000 platform. To monitor potential laboratory contamination, extraction blanks were included during DNA extraction and no‐template negative controls were included in each PCR. These controls were carried through the laboratory workflow together with the fecal samples. To ensure transparency and reproducibility, our reporting followed the latest Minimum Information for Environmental Metabarcoding (MIEM) guidelines (Klymus et al. 2024) and the FAIR data formatting standards for eDNA metadata (Takahashi et al. 2025), providing methodological details in Table S1.

#### 2.2.2 Dietary Species Identification

Raw sequencing data were subjected to quality control using fastp (v0.19.6) and assembly via FLASH (v1.2.7) before import into the QIIME2 analysis platform (Bolyen et al. 2019). The DADA2 algorithm (Callahan et al. 2016) was used for denoising to obtain high‐resolution amplicon sequence variants (ASVs). Taxonomic annotation was performed using different reference databases according to the markers: MitoFish (Release 3.75; http://mitofish.aori.u‐tokyo.ac.jp/) was used for the fish‐specific 12S sequences, while the NCBI nt (nucleotide) database (https://www.ncbi.nlm.nih.gov/nucleotide/; accessed October 2025) was used for the *COI* sequences.

Following species annotation, sequences that failed to be identified or that belonged to taxa unlikely to be tern prey were removed. The excluded sequences included mammals (humans and rodents), host species, bacteria, fungi, plants, and parasites (including Eimeria and Isospora). After these processing steps, and considering the study's focus on tern food sources, only species detected in Zhejiang were retained for subsequent analysis. Species distribution information was referenced from the marine fish in Zhejiang (Zhao et al. 2016). To assess whether the sample size was sufficient to represent the dietary results, species accumulation curves were constructed from filtered species ASV data using the vegan package in R (version 4.1.2; R Foundation for Statistical Computing, Vienna, Austria). For the *COI* marker, 27 out of 50 samples were successfully amplified via PCR. Following initial bioinformatic filtering to remove non‐target taxa, 16 samples were retained for further analysis. However, six of these samples were subsequently excluded due to insufficient sequencing depth (read counts below 20,000), leaving a final set of 10 samples for analysis.

Dietary data were analyzed pooled across all available samples using the following metrics: (1) occurrence frequency (FO, %), the percentage of samples in which a specific taxon was detected (number of samples containing the taxon/total number of samples); (2) Relative Read Abundance (RRA, %), the mean proportion of reads assigned to a specific taxon across all samples, providing an estimate of relative biomass contribution; and (3) Relative Frequency of Occurrence (RFO, %), the frequency of a specific taxon expressed as a percentage of the sum of all taxon detections across the dataset (number of samples containing the taxon/sum of occurrences of all taxa in all samples × 100) (Deagle et al. 2019). RRA‐based estimates can be influenced by PCR amplification bias (e.g., differential polymerase affinity) (Fonseca 2018), therefore, we presented these data with three parameters.

#### 2.2.3 Comparison Between Different Stages and Islands

After excluding samples that failed quality control or yielded insufficient DNA reads, a final set of 44 samples was used for these group comparisons. To investigate the potential influence of breeding phenology (i.e., the shift in physiological demands from egg production and incubation to chick provisioning) on dietary requirements, the data were analyzed using two grouping strategies: breeding period (breeding stage: *n* = 15, nestling stage: *n* = 29 as whole, and 9 vs. 27 in Xiangshan) and geographical region (Xiangshan Island: *n* = 36, Nanji Island: *n* = 8). Species accumulation curves showed that sampling for 12S was adequate (curve plateaued), while sampling for *COI* was insufficient. Given the lack of sufficient amplification efficiency of the *COI* primer, we used only the results from the fish 12S marker for subsequent analysis.

Intergroup comparative analyses were conducted as follows: alpha diversity indices were calculated using Mothur (v.1.48, a microbial ecology software suite). Specifically, the Chao1 index was used to estimate species richness (the number of different prey taxa), while the Shannon index reflected species diversity (accounting for both richness and the evenness of prey distribution). Due to the non‐normal distribution of our diversity data, the non‐parametric Wilcoxon rank‐sum test was employed to analyze differences between groups, with alpha diversity indices as response variables and breeding stages or geographical regions as explanatory variables. Principal coordinate analysis (PCoA) based on Bray‐Curtis distance and Jaccard distance was performed to examine similarities in dietary community structure between sample groups. Bioinformatic analysis of the sequencing data was performed using the online platform provided by Majorbio (https://www.majorbio.com/tools). All figures for data visualization were generated using ggplot2 package in R software.

### 2.3 Analysis of Tern Foraging Requirements Across the World

In this study, we summarized the vertical habitat distribution and economic attributes of the main prey consumed by breeding terns in Zhejiang. Dietary data from patterns in Australia (Chiaradia et al. 2002; Dunlop et al. 2025; McLeay et al. 2009) and South Africa (Gaglio et al. 2018) were compiled for comparison. We focused only on summarizing the main fish species detected with a relative abundance greater than 10% (defined as highly utilized by terns).

## 3 Results

### 3.1 Dietary Composition of Terns on Zhejiang Breeding Islands

Analysis of the amplicon sequencing data from the fish‐specific 12S rRNA region yielded 44 samples retained for downstream analyses. Based on field records, these comprised 5 Chinese crested tern and 39 greater crested tern samples. By location, 36 samples were from Xiangshan (9 from the breeding stage, 27 from the nestling stage) and 8 were from Nanji (6 from the breeding stage, 2 from the nestling stage). After quality filtering and denoising (details in Table S2), 427 ASV with 3,114,967 reads were obtained, and retained 341 ASV with 1,347,308 reads after filtering for prey. Analysis of the metazoan *COI* region amplicon sequencing data yielded 747 ASV with 1,426,993 reads after quality filtering and denoising, 97 ASV with 214,750 reads after filtering for prey. Only 10 samples were retained for downstream analyses (the remaining samples were removed as they contained no target prey or had insufficient sequencing depth). After resampling process, the following species annotation and filtering, 36 fish species were identified (belonging to 10 orders, 23 families, and 33 genera) (Table 2).

**TABLE 2:** Food sources of terns in Zhejiang, in family category.

Family	Habitat	Data sources	Occurrence frequency (%)	RFO (%)	RRA (%)	Species
Engraulidae	Marine	12S	72.73	34.04	43.98	*Thryssa kammalensis, Setipinna tenuifilis, Engraulis japonicus, Stolephorus insularis, Coilia mystus*
	Marine	COI	76.47	46.43	75.04	*E. japonicus, S. tenuifilis, Stolephorus commersonnii, T. kammalensis*
Scombridae	Marine	12S	27.27	12.77	10.03	*Scomber japonicus, Scomberomorus niphonius*
	Marine	COI	29.41	17.86	17.88	*S. japonicus, S. niphonius*
Clupeidae	Marine	12S	20.45	9.57	10.71	*Sardinella zunasi, Etrumeus teres*
Sciaenidae	Marine	12S	27.27	12.77	6.88	*Johnius belangerii, Collichthys lucidus, Larimichthys polyactis,Pennahia argentata*
Synodontidae	Marine	12S	13.64	6.38	6.66	*Saurida microlepis*
	Marine	COI	17.65	10.71	3.54	*Harpadon nehereus*
Gobionidae	Marine	12S	9.09	4.26	5.6	*Tridentiger bifasciatus*
Sparidae	Marine	12S	6.82	3.19	5.03	*Evynnis tumifrons*
	Marine	COI	5.88	3.57	1.95	*Evynnis cardinalis*
Cyprinidae	Freshwater	12S	2.27	1.06	2.66	*Cyprinus carpio*
Xenocyprididae	Freshwater	12S	2.27	1.06	2.66	*Megalobrama terminalis, Pseudolaubuca sinensis*
Centrolophidae	Marine	12S	2.27	1.06	2.39	*Psenopsis anomala*
Gobiidae	Marine	12S	9.09	4.26	2.38	*Tridentiger bifasciatus, Chaeturichthys stigmatias, Amblychaeturichthys hexanema*
	Marine	COI	5.88	3.57	0.18	*Chaeturichthys stigmatias*
Hemiramphidae	Marine	12S	2.27	1.06	0.45	*Hyporhamphus quoyi*
	Marine	COI	5.88	3.57	0.35	*Hyporhamphus regularis*
Sinipercidae	Freshwater	12S	2.27	1.06	0.27	*Siniperca obscura*
Carangidae	Marine	12S	6.82	3.19	F0.17	*Seriolina nigrofasciata, Decapterus maruadsi, Evynnis tumifrons*
Exocoetidae	Marine	12S	2.27	1.06	0.12	*Cheilopogon arcticeps*
	Marine	COI	5.88	3.57	0.18	*Hirundichthys coromandelensis*
Trichiuridae	Marine	12S	4.55	2.13	0.02	*Trichiurus japonicus*
Belonidae	Marine	12S	2.27	1.06	0.01	*Strongylura anastomella*
Dussumieriidae	Marine	COI	17.65	10.71	0.88	*Etrumeus teres*

*Note:* Habitat indicates the primary environment of the prey taxa (e.g., marine or freshwater). Data sources refer to the molecular markers used for taxonomic identification, specifically the 12S rRNA and COI gene regions. Occurrence frequency (%) represents the percentage of samples in which a specific prey family was detected. RFO (%) (Relative Frequency of Occurrence) was calculated as the frequency of one family divided by the sum of frequencies of all identified families. RRA (%) (Relative Read Abundance) denoted the proportion of sequencing reads assigned to a specific family relative to the total number of prey reads. Species listed the representative species identified within each corresponding family.

**FIGURE 2:**
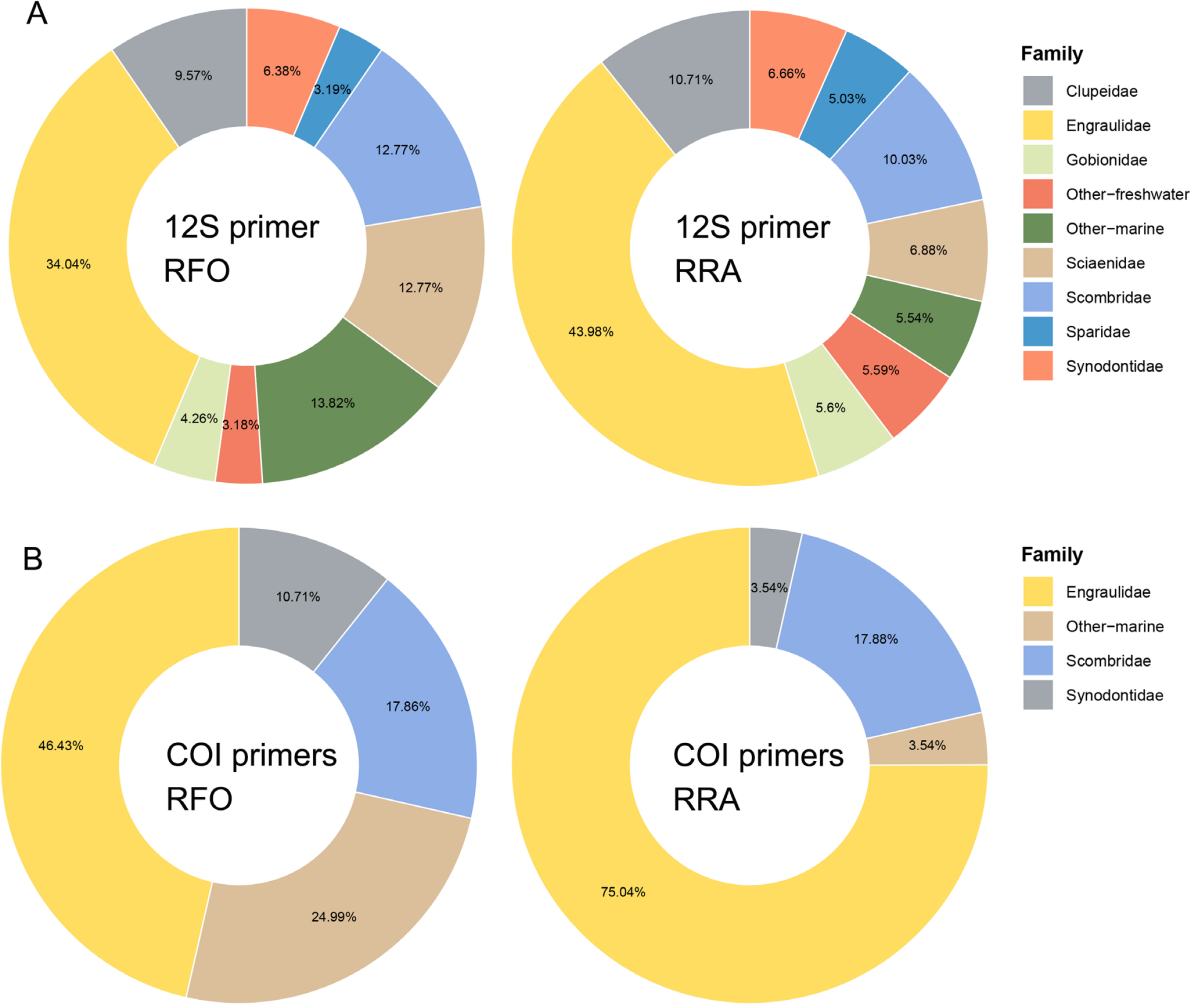
(A) Taxonomic composition of the tern diet based on (A) 12S and (B) COI metabarcoding. Data are pooled across all sampled species, breeding stages, and locations to provide an overall dietary profile. Families with a Relative Frequency of Occurrence (RFO) and Relative Read Abundance (RRA) below 3% are grouped into the “Others” category.

Based on the 12S rRNA data analysis for fish (Figure 2A), the top three families in terms of RRA were Engraulidae, Scombridae, and Clupeidae, with RRA values of 43.98%, 10.03%, and 10.71%, respectively. According to RFO, the top three families were Engraulidae, Scombridae, and Sciaenidae, with RFO values of 34.04%, 12.77%, and 12.77%, respectively. This indicated that Engraulidae and Scombridae were the primary dietary sources of terns during the breeding season in Zhejiang. The primary species identified within the family Engraulidae were *E. japonicus* (RFO = 14.66%, RRA = 12.28%), *Setipinna tenuifilis* (RFO = 11.21%, RRA = 12.94%), and *Thryssa kammalensis* (RFO = 11.21%, RRA = 15.38%). Within the family Scombridae (RFO = 12.77%, RRA = 10.03%), the predominant species were *Scomber japonicus* (RFO = 7.76%, RRA = 9.31%) and *Scomberomorus niphonius* (RFO = 2.29%, RRA = 0.57%). The other species with RRA and RFO values close to 10% were *Sardinella zunasi* (RFO = 5.17%, RRA = 9.98%). Low‐abundance detections of freshwater fish families (Xenocyprididae, Cyprinidae, and Sinipercidae) were identified in three individual samples, with each taxon appearing in only one sample (*n* = 1 per taxon).

Analysis based on *COI* data similarly showed the dominance of Engraulidae and Scombridae among fish (Engraulidae RFO = 46.43%, RRA = 75.05%; Scombridae RFO = 17.86%, RRA = 17.87%; Figure 2B). At the species level, the top three species identified by RRA were *T. kammalensis* , *S. tenuifilis* , and *S. japonicus* , whereas the top three Engraulidae species were *T. kammalensis* , *S. tenuifilis* , and *E. japonicus* . Among the arthropods, no species that could be confirmed as dietary components were detected.

### 3.2 Comparison of Tern Diets Across Breeding Stages and Islands in Zhejiang

#### 3.2.1 Comparison of Different Behavior Stages

**FIGURE 3:**
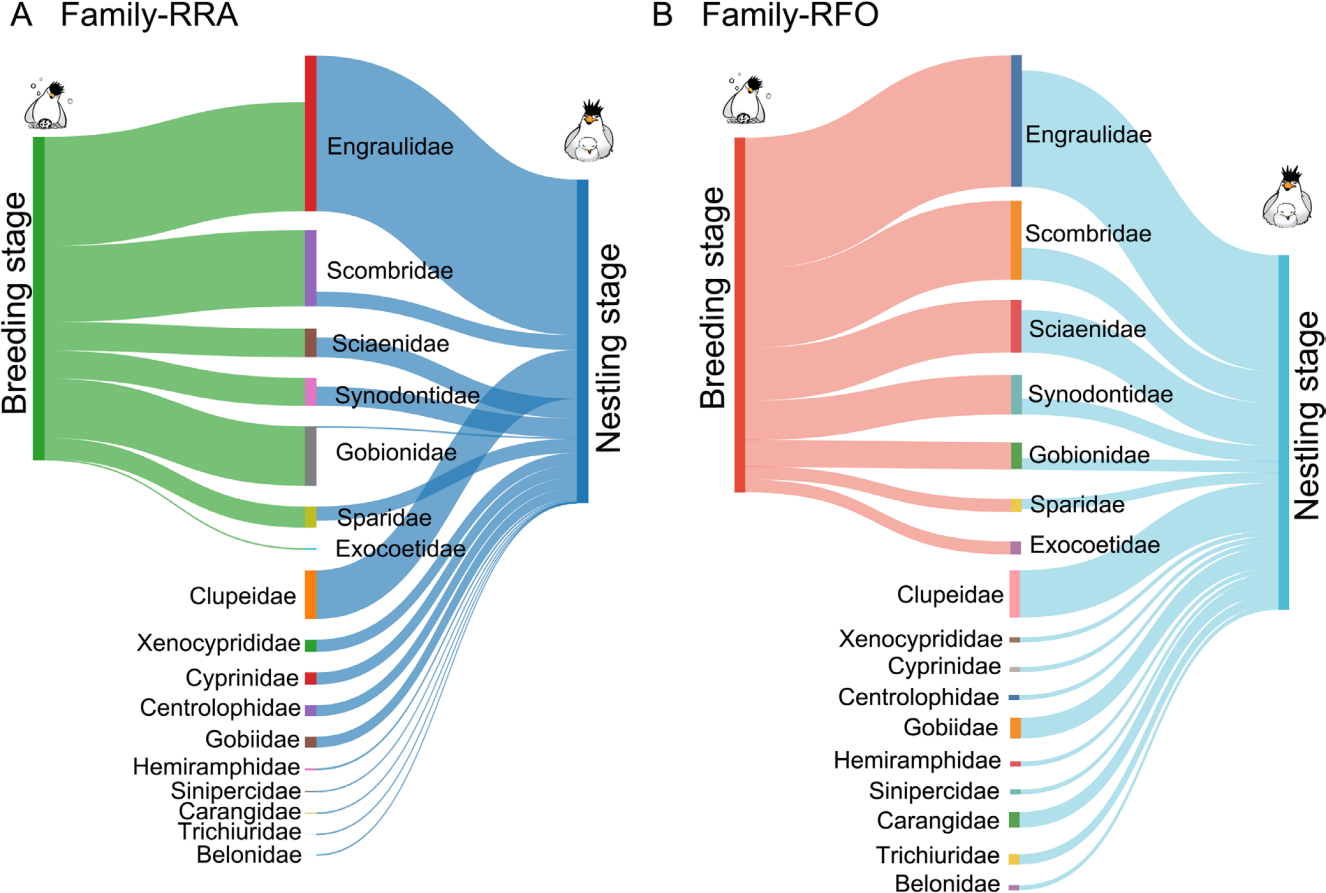
Comparison of tern diet composition across different periods at family levels. based on (A) RRA and (B) RFO.

In the temporal comparison between the breeding and nestling stages, the dietary composition of the terns exhibited distinct stage‐specific successional characteristics. The data indicated that while the primary food sources in both stages were dominated by Engraulidae, Sciaenidae, and Scombridae, the food sources during the nestling stage were more diverse (7 families vs. 16 families, Table 3, Figure 3A, Figure S1A). Furthermore, the detection rates of Clupeidae, Gobiidae, and Carangidae at the nestling stage exceeded 10%. Clupeidae was a prominent supplementary source (RFO = 13.44%, RRA = 15.06%).

**TABLE 3:** Dietary variation and alpha diversity of terns among different groups.

Group	Stage		Region	
	Breeding stage	Nestling stage	Nanji	Xiangshan
*N*	15	29	8	36
No. of prey families	7	16	6	17
Top three family (%RRA)	Engraulidae (33.74) Scombridae (23.51) Gobionidae (18.39)	Engraulidae (48.13) Clupeidae (15.06) Sciaenidae (6.10)	Scombridae (31.22) Engraulidae (27.23) Gobionidae (16.71)	Engraulidae (43.98) Clupeidae (10.71) Scombridae (10.03)
Top three family (%RFO)	Engraulidae (37.04) Scombridae (22.22) Sciaenidae (14.81)	Engraulidae (32.84) Clupeidae (13.44) Sciaenidae (11.94)	Engraulidae (33.33) Scombridae (28.57) Sciaenidae (23.81)	Engraulidae (34.04) Scombridae (12.77) Sciaenidae (12.77)
No. of prey species	13	29	9	28
Top three species (%RRA)	*Engraulis japonicus* (30.85) *Scomber japonicus* (23.51) *Abbottina rivularis* (13.15)	*Thryssa kammalensis* (21.58) *Setipinna tenuifilis* (18.19) *Sardinella zunasi* (14.04)	*S. japonicus* (31.22) *E. japonicus* (23.82) *A. rivularis* (16.71)	*T. kammalensis* (18.26) *S. tenuifilis* (15.38) *S. zunasi* (11.87)
Top three species (%RFO)	*E. japonicus* (29.03) *S. japonicus* (19.35) *Saurida microlepis* (9.68)	*T. kammalensis* (14.12) *S. tenuifilis* (14.12) *E. japonicus* (9.41)	*S. japonicus* (26.09) *E. japonicus* (26.09) *Collichthys lucidus* (21.74)	*T. kammalensis* (12.90) *S. tenuifilis* (12.90) *S. zunasi* (11.83)
Alpha diversity				
Chao	**6.93**	**10.55**	9.44	8.75
Shannon	0.72	0.93	0.88	0.76
Simpson	0.62	0.55	0.56	0.59

*Note:* *N* represents the number of analyzed individual samples in each group. RRA (%) (Relative Read Abundance) was the proportion of sequencing reads assigned to a specific taxon relative to the total prey reads. RFO (%) (Relative Frequency of Occurrence) denoted the frequency of a taxon relative to the sum of frequencies of all identified taxa. Alpha diversity indices include Chao1 (species richness), Shannon (species diversity and evenness), and Simpson (species dominance). The bold comparison was with *p* < 0.05.

At the species level, the diet during the breeding stage was primarily dominated by *E. japonicus* and *S. japonicus* , with RRA of 30.85% and 23.51%, respectively (Figure S2A). *E. japonicus* also had the highest frequency of occurrence at this stage (29.03%). Additionally, *Abbottina rivularis* contributed notably to dietary component (RRA = 13.15%). However, a significant shift in the dominant prey occurred upon entering the nestling stage. Species within the family Engraulidae, namely *T. kammalensis* and *S. tenuifilis* , became the core prey, with RRA values of 21.58% and 18.19%, respectively (Figure 3B). Both species exhibited the highest frequency of occurrence during the nestling stage (14.12% each). The RRA of *S. zunasi* increased to 14.04%.

**FIGURE 4:**
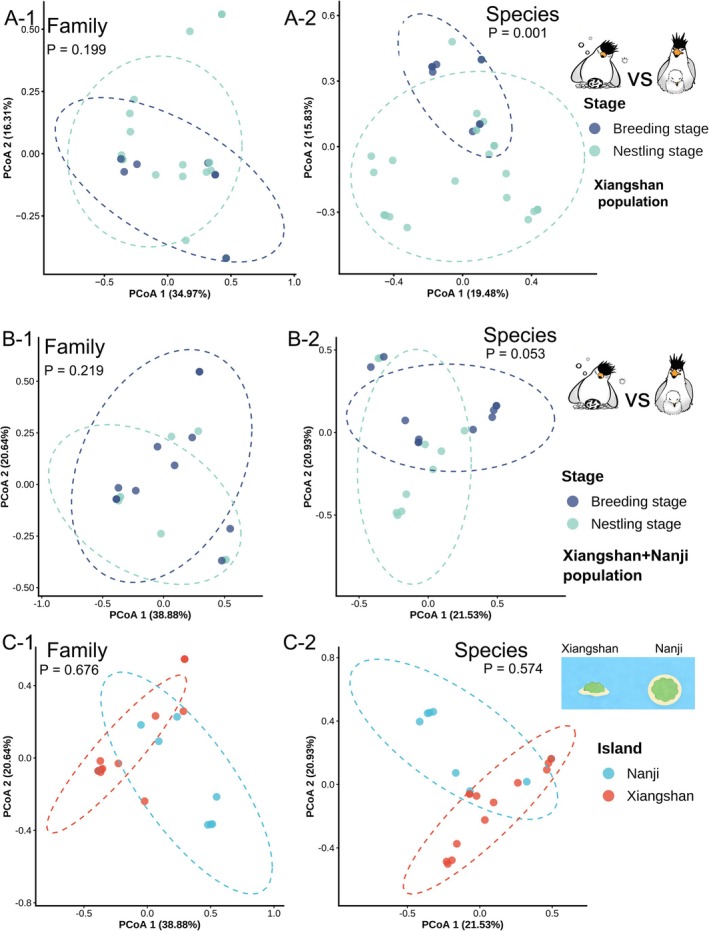
Principal coordinate analysis (PCoA) of tern diet communities based on Bray‐Curtis distance, showing results at the Family (1) and Species (2) levels for: (A) dietary shifts between breeding stages restricted to the Xiangshan population, (B) comparisons between breeding stages with pooled data from Xiangshan and Nanji populations, and (C) comparisons between different islands.

Analysis of dietary community structure revealed that at the family level, no significant differences were observed between stages. At the species level, while inter‐island variation remained nonsignificant (Figure 4C), significant differences between breeding stages were detected when focusing specifically on Xiangshan Island individuals (Figure 4A), and this result was further corroborated by our Jaccard distance‐based analysis (Figure S3A). When data from both islands were pooled, these stage‐related differences at the species level became nonsignificant (Figure 4B). Regarding alpha diversity, the Chao index was significantly higher in the nestling group (10.55 vs. 6.93, Table 3), indicating an expansion in prey species richness during the chick‐rearing period. This suggests that the terns may adopt a more opportunistic foraging strategy to meet the increased nutritional demands of their offspring. This increase in richness did not extend to other alpha diversity measures, as Shannon and Simpson indices showed no significant variation between groups.

#### 3.2.2 Comparison Across Different Breeding Islands in Zhejiang

A geographic comparison between breeding colonies on the Xiangshan Islands and Nanji Islands revealed that the Nanji groups utilized a narrower range of fish resources (6 families vs. 17 families). Nonetheless, the dominant prey for terns at both sites was concentrated in the families Engraulidae, Scombridae, and Sciaenidae.

At the species level, *T. kammalensis* and *S. tenuifilis* were the most dominant prey species on the Xiangshan Islands, with RRA of 18.26% and 15.38%, respectively. Both species were most frequently detected, appearing in 12 samples. *S. zunasi* was also suggested as a major dietary component (RRA = 11.87%, RFO = 11.83%). In contrast, the dominant prey structure in the Nanji group was distinct, characterized by a high reliance on *S. japonicus* and *E. japonicus* . Their RRA values were 31.22% and 23.82%, respectively, and both were detected in six samples (FO = 75% each). *A. rivularis* and *Collichthys lucidus* also held important positions in the Nanji diet, with RRAs of 16.71% and 12.85%, respectively. No significant differences in the dietary community were observed between the Nanji and Xiangshan groups. This conclusion holds true for both the overall community structure in both family and species level (Beta diversity, Figure 4C and Figure S2C) and within‐community diversity (Alpha diversity), as assessed by richness (Chao, Sobs) and evenness (Shannon, Simpson) indices (Table 3).

### 3.3 Vertical Distribution and Economic Attributes of Prey

Our study revealed that the dominant prey (RRA > 10%) of terns in Zhejiang were predominantly epipelagic or mid‐water fish species (Table 4). Among these, four of the five highly utilized fish primarily inhabited the epipelagic zone. The sole high‐abundance prey species occupying a different ecological niche is *S. tenuifilis* , a demersal species. Regarding commercial importance, several target species within the Xiangshan protected area were identified in the diet, including *C. lucidus* , *Johnius belangerii* , and *Larimichthys polyactis* . However, these species represented a minor portion of the overall diet, with the highest RRA value reaching only 4.63%.

**TABLE 4:** Terns' food sources information of our study and published data. The abundance in our study was using RRA, and percentage frequency in other studies.

Location	Tern species	Data sources and analysis method	Family	Species	Abundance	Habitat
Zhejiang	Chinese crested tern and greater crested tern	This Study, DNA metabarcoding	Engraulidae	*Engraulis japonicus*	30.90%	Epipelagic zone
			Scombridae	*Scomber japonicus*	23.50%	Epipelagic zone
			Engraulidae	*Thryssa kammalensis*	21.60%	Epipelagic zone
			Engraulidae	*Setipinna tenuifilis*	18.20%	Demersal
			Clupeidae	*Sardinella zunasi*	14.00%	Epipelagic zone
Australia	Greater crested tern	Dunlop et al. (2025), digital photography	Clupeidae	*Hyperlophus translucidus*	17.26%	Diel vertical migrate
			Clupeidae	*Sardinops sagax*	14.41%	Epipelagic zone
			Clupeidae	*Spratelloides robustus*	19.47%	Epipelagic zone
			Engraulidae	*Engraulis australis*	11.67%	Epipelagic zone
		Chiaradia et al. (2002), regurgitate analysis	Engraulidae	*E. australis*	54.50%	Epipelagic zone
			Carangidae	*Trachurus* spp.	25.50%	Epipelagic zone
			Gempylidae	*Thyrsites atun*	6.50%	Epipelagic zone
		McLeay et al. (2009), regurgitate analysis	Monacanthidae	*Thamnaconus degeni* ^a^	35.30%	Demersal
			Clupeidae	*Sardinops sagax* ^a^	17.00%	Epipelagic zone
			Engraulidae	*Engraulis australis* ^b^	13.10%	Epipelagic zone
South Africa	Greater crested tern	Gaglio et al. (2018), digital photography	Engraulidae	*Engraulis capensis*	69.96%	Epipelagic zone
			Scomberesocidae	*Scomberesox saurus*	5.80%	Epipelagic zone
			Clupeidae	*Etrumeus whiteheadi*	8.34%	Epipelagic zone

*Note:* ^a^The event was happened in 2005 and 2006; ^b^in 2007.

## 4 Discussion

This study utilized DNA metabarcoding to analyze the dietary composition and prey diversity of the Chinese crested tern and greater crested tern on breeding islands in Zhejiang Province. The results identified Engraulidae and Scombridae as the primary food sources for terns during the breeding season in Zhejiang. At the species level, the main prey species within Engraulidae were *E. japonicus* , *S. tenuifilis* , and *T. kammalensis* ; within Scombridae, the dominant species included *S. japonicus* and *S. niphonius*. These taxa represent key epipelagic forage fish and mid‐trophic functional groups, which are essential for energy flow in the East China Sea ecosystem. Their dominance in the diet, also confirmed by our field surveys (Figure 1B), underscores the terns' reliance on these specific resources for breeding success. Dietary variations were observed between breeding stages (breeding vs. nestling), with the nestling stage being characterized by a greater diversity of prey sources, as supported by the Chao index. Comparisons between different islands revealed that while the prominent prey families were similar, the relative contributions of dominant prey species differed. This research provides a foundation for a deeper understanding of tern conservation on breeding islands in the Zhejiang Province.

### 4.1 Stable Foraging Strategies and Regional Dietary Plasticity for Terns

According to the previous research (Burke and Montevecchi 2009), seabirds function as central foragers during the breeding season, and the distance from their breeding sites limits their distribution. This means that the abundance of food resources in surrounding marine areas directly influences population numbers. However, our results show a clear mismatch between environmental prey availability and actual consumption. While various fish species dominate the local marine community (Table S3), only *S. tenuifilis* was the most consistently detected taxon, accounting for a substantial proportion of the total sequence reads (RRA > 10%). This discrepancy indicates that terns do not forage based solely on total abundance but exhibit high selectivity for epipelagic prey, as many locally dominant species are non‐pelagic and thus less accessible due to the restricted diving depth and surface‐foraging strategy of terns.

Cross‐regional comparisons (Table 4) reveal that the preference for epipelagic schooling fish is not a localized phenomenon in Zhejiang, but represents a stable, species‐level foraging strategy for the greater crested tern. While this overarching strategy remains consistent across its range, the taxonomic composition is highly plastic, reflecting regional prey availability and accessibility. In Zhejiang, terns primarily exploit locally abundant species such as *E. japonicus* , *S. japonicus* , and *T. kammalensis* . Similarly, Australian populations rely on epipelagic fish like *Engraulis australis* and *Sardines sagax* (Chiaradia et al. 2002; McLeay et al. 2009; Dunlop et al. 2025), while South African diets are overwhelmingly dominated (69.96%) by *Engraulis capensis* (Gaglio et al. 2018).

Interestingly, the inclusion of certain prey reflects the terns' ability to exploit vertical resource shifts. For instance, *Hyperlophus translucidus* exhibits diel vertical migration, becoming accessible at the water surface during dawn or dusk. Even when atypical prey are recorded—such as the high proportion of the demersal fish *Thamnaconus degeni* in Australia (McLeay et al. 2009)—this likely reflects localized foraging on juveniles that have temporarily ascended to surface waters. Collectively, these data demonstrate that the greater crested tern's diet is a product of specialized predatory traits filtered through the lens of regional abundance and the vertical availability of resources near breeding sites.

### 4.2 Dietary Insights for the Conservation of Breeding Terns in Zhejiang

**FIGURE 5:**
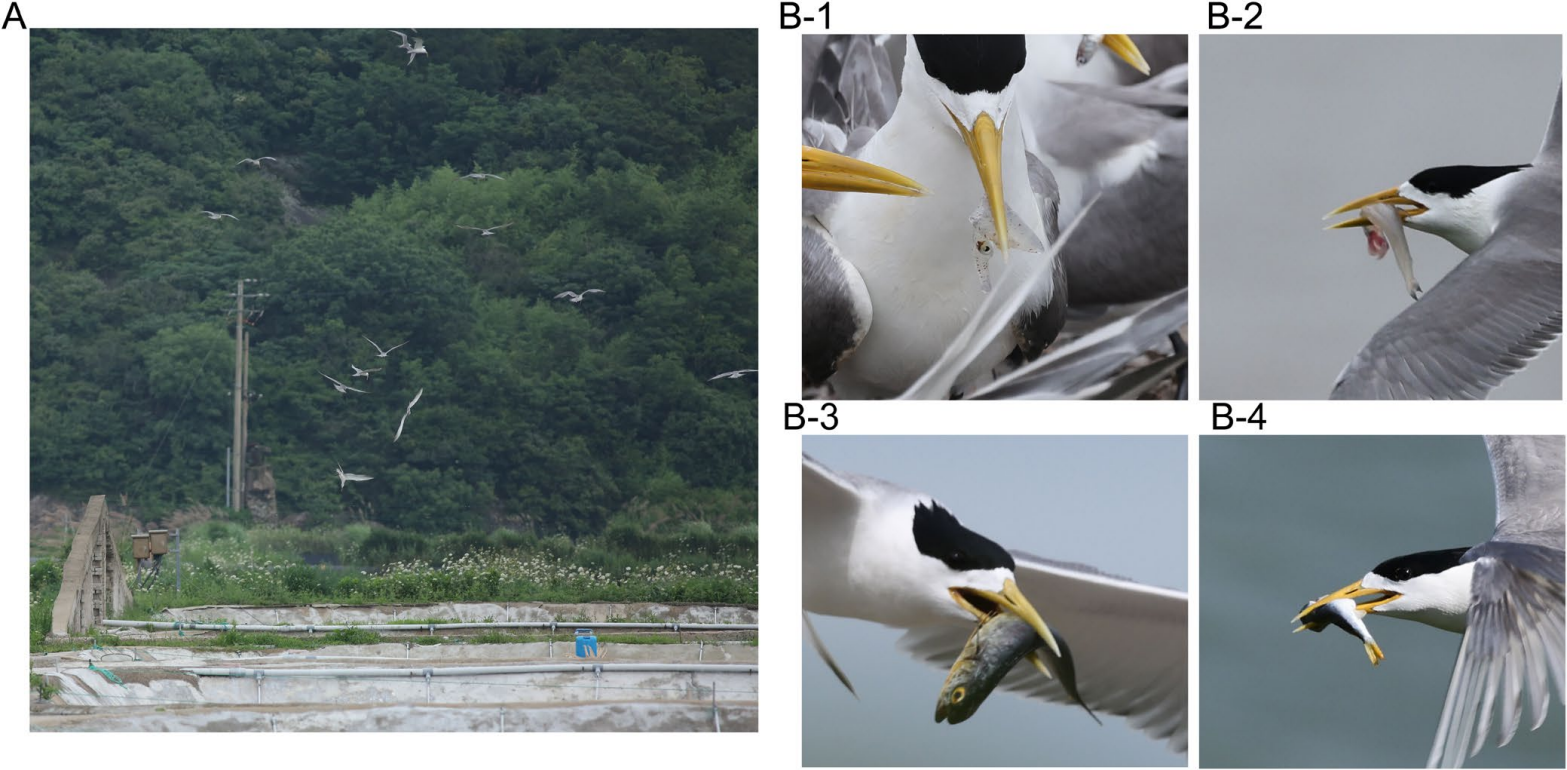
(A) Field observation of terns foraging in an inland aquaculture pond and (B) undetected dietary species recorded in the field monitoring. (B1) Squid, (B2) *Harpadon*, (B3) *Coryphaena hippurus* , and (B4) *Takifugu xanthopterus* . Photo credit: Siyu Wang.

Given that Zhejiang coastal waters host half of the world's breeding sites for the critically endangered Chinese crested tern (Wang et al. 2026), understanding their dietary requirements is essential for maintaining the habitat's carrying capacity. Our study provides insights into the potential relationship between the diet of terns and local fishery resources. Although the waters around the Xiangshan Islands serve as important spawning and nursery grounds for various commercial fish species (such as *Trichiurus japonicus* , *L. polyactis* , *Nibea albiflora* , and *Miichthys miiuy* ), they consume these species in very low proportions (RRA < 5%), primarily feeding on small epipelagic fishes, such as the Engraulidae family. Notably, demersal fish such as *S. tenuifilis* constitute a relatively high proportion of the diet of terns (18.20%). This likely reflects an expansion of their nearshore foraging behavior rather than direct pressure on commercial fishery resources. Therefore, ensuring the sustainability of key forage fish resources, particularly those in the Engraulidae family, during the pre‐breeding period should be a focal point for regional management. Although these small pelagic fish are not always the primary targets of large‐scale commercial operations, they are sensitive to intensive regional fishing pressure which has led to a documented decline in biomass (Cheng et al. 2009). To better protect these resources, management actions should include incorporating Engraulidae into routine fishery resource monitoring and ensuring that the summer fishing moratorium effectively covers the peak breeding window of the terns. Such measures would help maintain a stable prey base during this critical life stage, bridging the gap between general fishery policy and specific species conservation. However, some food resources recorded during the field monitoring were not detected in this study (Figure 5B). This indicates that extensive monitoring is required to obtain comprehensive data.

The detection of freshwater fish DNA (total FO = 6.82%) provides empirical evidence of dietary plasticity in these terns. While their primary niche is marine, the utilization of aquaculture ponds and inland rivers (Figure 5A) exposes them to novel anthropogenic threats at the land‐sea interface. Although specific capture events were not visually recorded during our site surveys, the integration of molecular evidence and field observations (Figure 5A) confirms that these anthropogenic inland habitats serve as integral parts of the terns' broader foraging strategy. Given this documented behavior, conservation management must address risks associated with coastal aquaculture, such as promoting bird‐friendly practices to prevent incidental injury from anti‐bird nets.

### 4.3 Study Limitations

While DNA metabarcoding identified primary prey, several limitations exist. Nevertheless, this approach has proven effective in conservation of other threatened avian species, including the wintering *Grus japonensis* (Liu et al. 2023). The sample size (*n* = 44) primarily reflects dominant dietary components. The absence of marine invertebrates in this specific analysis likely stems from biological factors rather than technical bias; notably, our concurrent COI analysis of chick samples successfully detected arthropods (unpublished data), suggesting that the lack of invertebrates in other samples may reflect rapid DNA degradation in adult digestive tracts. Additionally, the limited sample size of Chinese crested terns individuals precluded a detailed interspecific niche comparison. Furthermore, while pooling samples provides a population‐level overview, it may overlook subtle interspecific niche differences. Future work should integrate quantitative frameworks like (Shelton et al. 2023) to correct for PCR bias; such models are vital for evolving from read counts to precise biomass estimates.

## 5 Conclusion

Our study demonstrates that breeding terns in Zhejiang are specialized predators primarily reliant on small epipelagic forage fish, particularly from the family Engraulidae, while exhibiting opportunistic use of freshwater resources. These results suggest that ensuring breeding success depends less on the management of large commercial stocks and more on the stability of key forage fish populations throughout the breeding season. Future conservation strategies should prioritize the long‐term monitoring of Engraulidae resources and the mitigation of anthropogenic risks in coastal foraging habitats, thereby moving from simple nesting site protection to a more comprehensive strategy ensuring foraging security for these critically endangered seabirds.

## Author Contributions

**Peng Ding:** methodology (equal), resources (equal), writing – original draft (equal). **Xilai Zhou:** investigation (equal), methodology (equal), software (equal). **Siyu Wang:** conceptualization (equal), investigation (equal), resources (equal). **Yiwei Lu:** data curation (equal), investigation (equal). **Zhiwen Yan:** data curation (equal), investigation (equal). **Cheng Qian:** data curation (equal), investigation (equal). **Keying Zhu:** data curation (equal). **Xiaopin Ni:** data curation (equal), investigation (equal). **Erwei Wu:** data curation (equal), investigation (equal). **Zhongyong Fan:** conceptualization (equal), data curation (equal), funding acquisition (equal), project administration (equal), resources (equal), writing – original draft (equal). **Ke He:** conceptualization (equal), data curation (equal), formal analysis (equal), resources (equal), software (equal), visualization (equal), writing – original draft (equal), writing – review and editing (equal).

## Funding

The project was supported by Key R&D Program of Zhejiang Province (No. 2021C02044), National Natural Science Foundation of China (No. 32370545), and Zhejiang Rare and Endangered Wildlife Rescue and Conservation Project of Zhejiang Provincial Forestry Bureau (2021–2025).

## Conflicts of Interest

The authors declare no conflicts of interest.

## Supporting information


**Figure S1:** Comparison of tern diet composition across (A) different periods and (B) different islands.

**Figure S2:** Comparison of tern diet composition across different periods at species levels based on (A) RRA and (B) RFO. RRA below 3% were excluded from the species‐level comparison.

**Figure S3:** Principal coordinate analysis (PCoA) of tern diet communities based on Jaccard distance, showing (A) dietary shifts between breeding stages restricted to the Xiangshan population, (B) comparisons between breeding stages with pooled data from Xiangshan and Nanji populations, and (C) comparisons between different islands.

**Table S1:** Reporting checklist of DNA metabarcoding in this study.
**Table S2:** Summary of sequence filtering.
**Table S3:** Summary of dominant fish species in surrounding waters of Xiangshan Island based on regional fishery surveys.

## Data Availability

The raw sequence data have been deposited in the Genome Sequence Archive in the National Genomics Data Center, China National Center for Bioinformation/Beijing. Institute of Genomics, and Chinese Academy of Sciences under accession number CRA037280.
