# Supplementary figures and images for: DNA Metabarcoding Reveals the Diet and Conservation Needs of the Chinese Crested Tern and Greater Crested Terns in Zhejiang Breeding Islands
Source: Ecol Evol. 2026 Apr 16;16(4):e73492. doi: 10.1002/ece3.73492 (PMC13087090; doi:10.1002/ece3.73492)

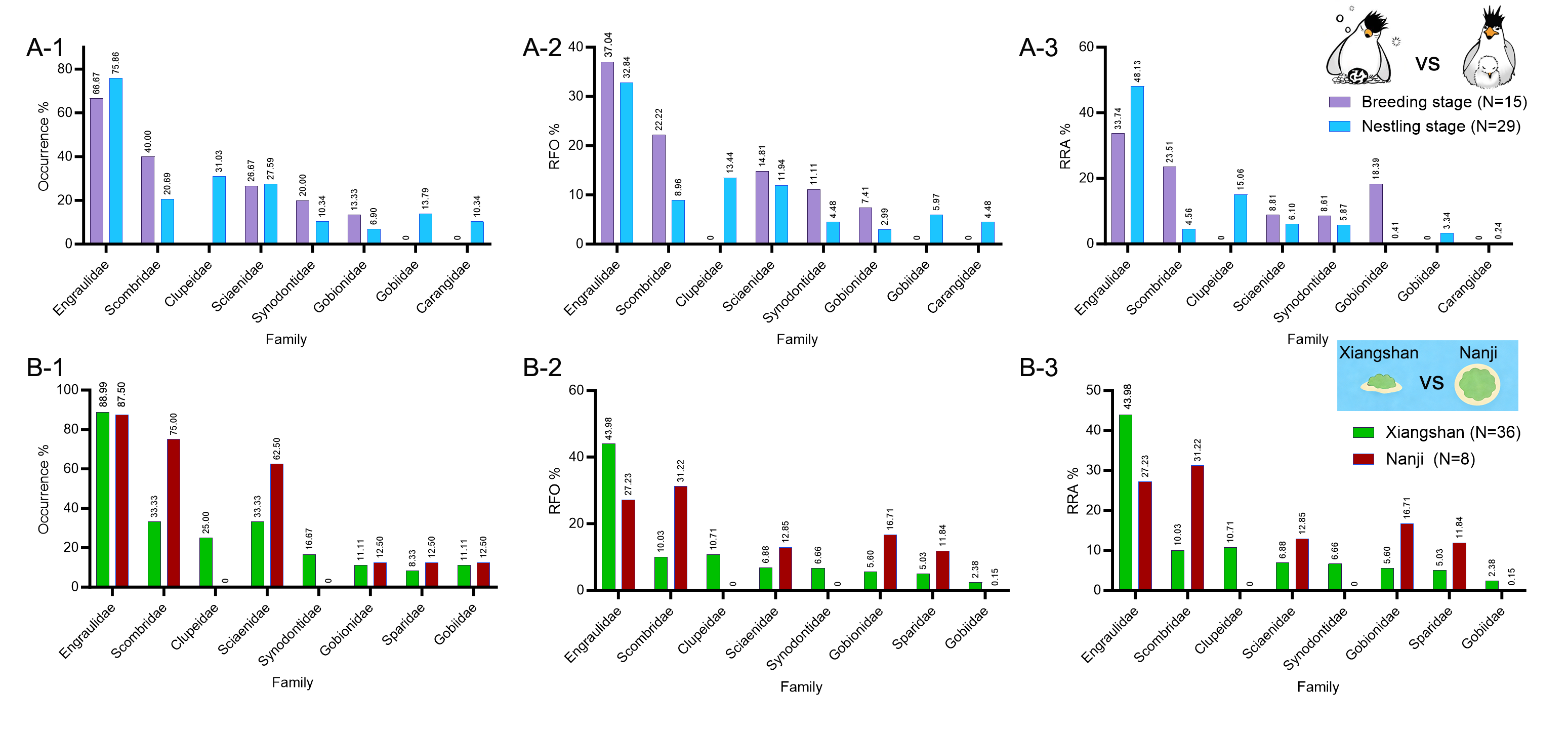

Supplement: Supplementary file 1 — Figure S1: Comparison of tern diet composition across (A) different periods and (B) different islands. [file ECE3-16-e73492-s002.tif]

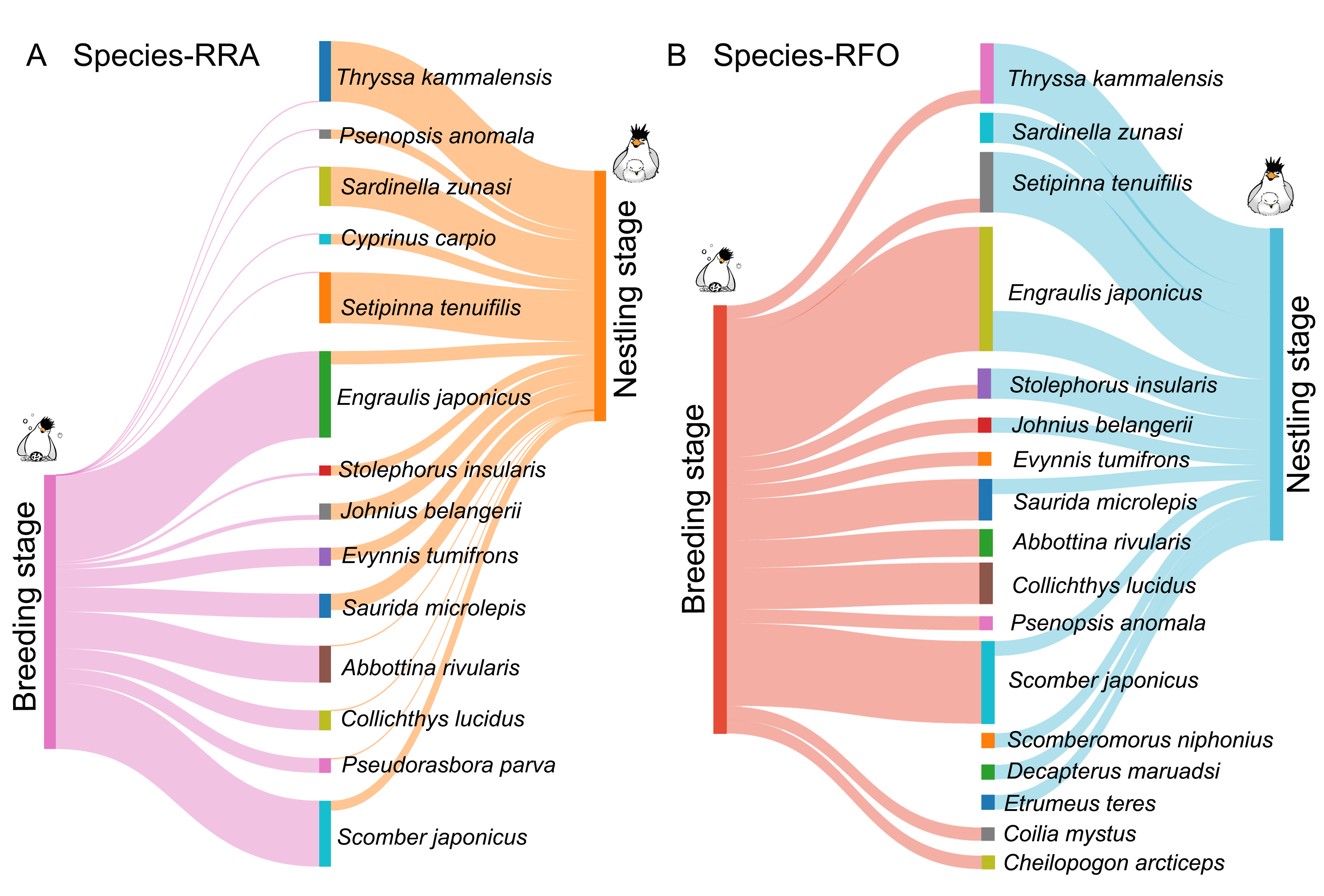

Supplement: Supplementary file 2 — Figure S2: Comparison of tern diet composition across different periods at species levels based on (A) RRA and (B) RFO. RRA below 3% were excluded from the species‐level comparison. [file ECE3-16-e73492-s001.tif]

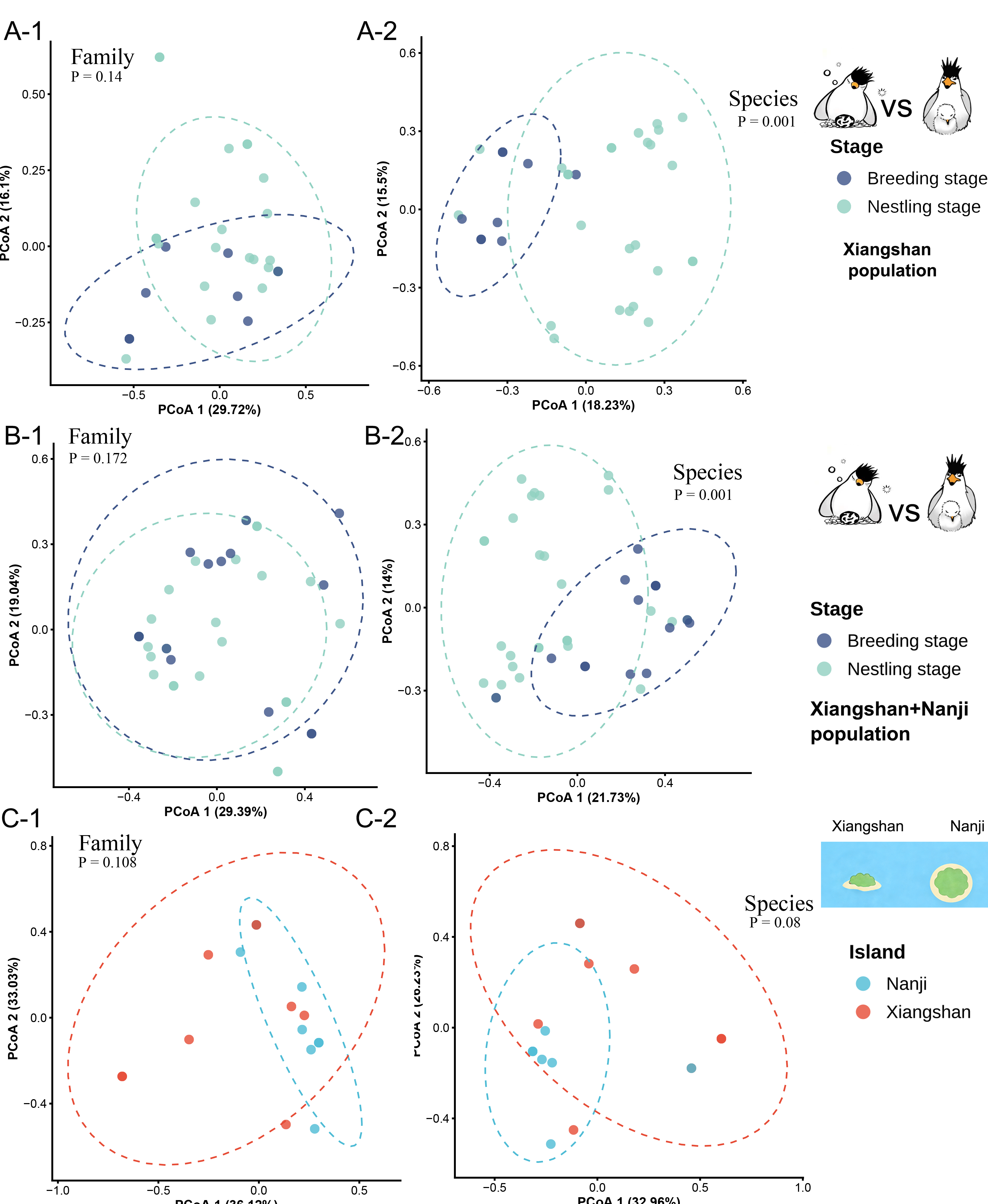

Supplement: Supplementary file 3 — Figure S3: Principal coordinate analysis (PCoA) of tern diet communities based on Jaccard distance, showing (A) dietary shifts between breeding stages restricted to the Xiangshan population, (B) comparisons between breeding stages with pooled data from Xiangshan and Nanji populations, and (C) comparisons between different islands. [file ECE3-16-e73492-s004.tif]
